# Complete mitochondrial genome and phylogenetic position of the Taranetz charr *Salvelinus taranetzi* Kaganovsky, 1955 (Salmoniformes: Salmonidae)

**DOI:** 10.1080/23802359.2019.1637292

**Published:** 2019-07-12

**Authors:** Alla G. Oleinik, Lubov A. Skurikhina, Andrey D. Kukhlevsky, Alexander A. Semenchenko

**Affiliations:** a A. V. Zhirmunsky Institute of Marine Biology, National Scientific Center of Marine Biology, Far Eastern Branch, Russian Academy of Sciences, Vladivostok, Russia;; b Far Eastern Federal University, Vladivostok, Russia

**Keywords:** charr genus *Salvelinus*, Taranetz charr *Salvelinus taranetzi*, Arctic charr *Salvelinus alpinus*, mtDNA, phylogeny

## Abstract

The complete mitochondrial genome was sequenced in two individuals of Taranetz charr *Salvelinus taranetzi*. The genome sequences are 16,654  bp in size, and the gene arrangement, composition, and size are very similar to the charr genomes published previously. The difference between the two genomes studied is low, 0.05%. The present study confirms the independent taxonomic status of *S. taranetzi* within the genus *Salvelinus*. The level of sequence divergence between *S. taranetzi* and *Salvelinus alpinus* inferred from the complete mitochondrial genomes is relatively low (1.1%), indicating recent divergence of the species.

The Taranetz charr *Salvelinus taranetzi* was described by Kaganovsky (1955) from Lake Achchen in the Chukotka Peninsula, Russia. Originally described as a separate species, it was subsequently synonymized with Arctic charr *Salvelinus alpinus taranetzi* (Behnke 1984). However, Glubokovsky and Chereshnev (Glubokovsky and Chereshnev 1981; Chereshnev 1982) pointed out that *S. taranetzi* is different from *S. alpinus* and may be regarded as a separate species. However, there is still a lack of molecular data to prove this opinion (Oleinik et al. 2017). Most of the previous studies of *S. taranetzi* along with other charr are restricted to an analysis of only short fragments of few mitochondrial and nuclear genes (e.g. Osinov et al. 2017, and references therein).

We sequenced and described two complete mitochondrial genomes of *S. taranetzi* for the first time in this study, for further study and more precise phylogenetic analysis. Specimens of Taranetz charr were collected from Lake Achchen, Chukchi Peninsula (64°50′ N, 174°36′ W), and Lake Pekulineiskoe, Chukchi Peninsula (62°33′ N, 177°17′ E); one specimen was collected from the type locality. The fish specimens are stored in the collection of the Genetics Laboratory, National Scientific Center of Marine Biology FEB RAS, Vladivostok, Russia (www.imb.dvo.ru). Totally 5 pairs of primers were used (sequences are available upon request), which were designed based on public sequences available in GenBank for salmonid fishes. The sequenced fragments were *de novo* assembled into complete mitochondrial genome and annotated by comparing with published genome sequences of charr using Geneious R11 (http://www.geneious.com/).

The complete mitochondrial genome of *S. taranetzi* was 16,654 bp in length (GenBank accession numbers MK695630 and MK695631). The genome organization was identical to that of typical salmon mitochondrial genomes. Similar to charr mitochondrial genomes (Balakirev et al. 2016), the overall base composition was 28.0% of A, 26.4% of T, 28.6% of C, and 17.0% of G with a slight A + T bias (54.5%). We detected 18 single-nucleotide and no length differences between the sequences MK695630 and MK695631; two substitutions were detected in the control region and 12S rRNA, other single-nucleotide substitutions were found in overall protein-coding sequences. The proportion of variable sites was highest for the NADH dehydrogenase subunit genes (55.6%). Total sequence divergence (*D_xy_*) was 0.0011 ± 0.0002.

The comparison of mitochondrial genomes now obtained with mitochondrial genomes of related groups available in GenBank including genera *Salvelinus* (AF154851, KF974451, KJ746618, KJ746619, KT266870, KT266871, KU674351, KU674352, NC000860, NC036392, and NC037502), *Salmo* (AF133701, and AM910409), and *Parahucho* (KJ816315, and KJ816316) point to the independent taxonomic status of *S. taranetzi* within the genus *Salvelinus* (Figure 1). The level of divergence (*Dxy*) between *S. taranetzi* and taxa within the phylogenetic group was in the range from 0.0095 ± 0.0006 to 0.0442 ± 0.0019. *Salvelinus taranetzi* specimens showed similar sequence divergence (0.0103 ± 0.0007 on average) from *S. malma malma*, *S. malma kuznetzovi*, *S. albus*, and *S. alpinus*. These values corresponded to the level of intraspecific variability in the genus, and all charr taxa showed similar phylogenetic relationships to those found by Oleinik et al. (2015).

**Figure 1.**
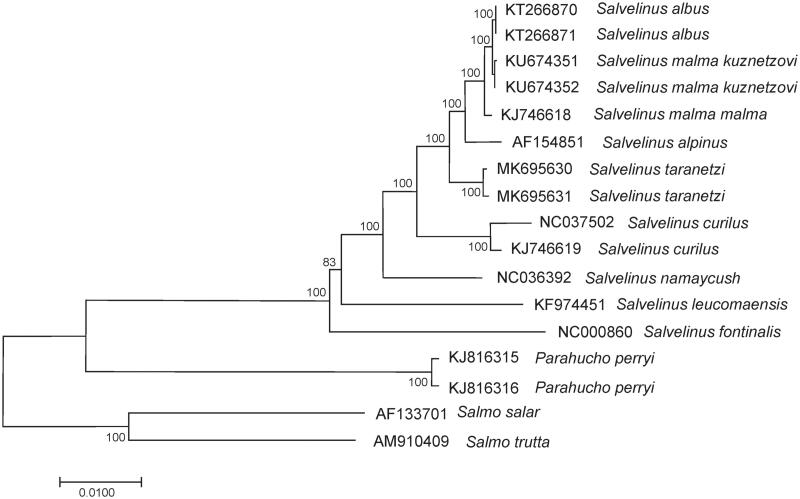
Maximum likelihood (ML) tree constructed based on the comparison of complete mitochondrial genome sequences of *Salvelinus taranetzi* and other GenBank representatives of the family Salmonidae. The tree is based on the TrN93 plus gamma model of nucleotide substitution. Genbank accession numbers for all sequences are listed in the figure. Numbers at the nodes indicate bootstrap probabilities from 1000 replications (values below 80% are omitted).
